# Predicting the Emergency Department Patient Journey Using a Machine Learning Approach

**DOI:** 10.2196/67321

**Published:** 2025-12-19

**Authors:** Joshua George Kovoor, Gavin John Carmichael, Brandon Stretton, Aashray K Gupta, Oliver S Kleinig, Mana Ittimani, Jack Fabian, Sheryn Tan, Jeng Swen Ng, Shrirajh Sateakeerthy, Andrew Booth, Alexander Beath, John Kefalianos, Mathew Ollapallil Jacob, Sadeya Ahmed, WengOnn Chan, Pramesh Kovoor, Samuel Gluck, Toby Gilbert, James Malycha, Benjamin A Reddi, Robert T Padbury, Markus I Trochsler, Guy J Maddern, Derek P Chew, Andrew C Zannettino, Danny Liew, John F Beltrame, Patrick G O’Callaghan, Cynthia Papendick, Stephen Bacchi

**Affiliations:** 1 Grampians Health, 1 Drummond Street N, Ballarat Central, 3350, Australia, 61 53204000; 2 The University of Melbourne, Grattan Street, Parkville, Australia; 3 The University of Adelaide, Adelaide, Australia; 4 New South Wales Health, Sydney, Australia; 5 Central Adelaide, Government of South Australia, Adelaide, Australia; 6 South Australia Health, Adelaide, Australia; 7 Westmead Hospital, Sydney, Australia; 8 The Queen Elizabeth Hospital, Adelaide, Australia; 9 Flinders University, Adelaide, Australia; 10 Royal Adelaide Hospital, Adelaide, Australia

**Keywords:** AI, artificial intelligence, prediction, TF-IDF, health informatics, patient care, hospital resource management, resource management, language model, machine learning, hospitalization, deep learning, logistic regression, retrospective analysis, large language model, emergency department

We were pleased to read Patel et al’s article, “Traditional Machine Learning, Deep Learning, and BERT (Large Language Model) Approaches for Predicting Hospitalizations from Nurse Triage Notes” [1] published in *JMIR AI*. The study compared machine learning (ML) models, including the Bidirectional Encoder Representations from Transformers (BERT)–based model “Bio-Clinical-BERT” and term frequency–inverse document frequency (TF-IDF), to predict hospitalizations based on nurse triage notes. We commend the authors for their valuable contribution to the field of ML predictive analytics. Their findings align with our recent work aimed at enhancing patient flow through emergency departments (ED) using ML models. We wish to highlight our study to further contribute to this growing body of research.

We aimed to evaluate the performance of various ML models in predicting three key outcomes in ED patients’ journeys: prolonged ED length of stay (LOS ≥8 h), chest x-ray (CXR) utilization, and inpatient admissions. We analyzed data from 50,000 ED visits at two major public metropolitan hospitals in South Australia and tested XGBoost (extreme gradient boosting), random forest, and logistic regression models. Our primary objective was to assess model accuracy in predicting the outcomes to support clinical decision-making and enhance operational efficiency.

The patient cohort had a mean age of 52.5 (SD 22.1) years (25,211/50,000, 50.4% female). Additionally, 78.6% (n=39,300) of patients reported English as their primary language. Median ED LOS was 4 hours 31 minutes (IQR 2 h 50 min to 7 h 8 min). CXRs were ordered for 27.2% (n=13,578) of patients, and 26.7% (n=13,343) were admitted as inpatients.

Among the models evaluated, XGBoost demonstrated the strongest performance across all predictive tasks, achieving area under the receiver operating characteristic curve (AUROC) values of 0.79 for predicting prolonged ED LOS, 0.88 for CXR utilization, and 0.85 for inpatient admissions. The random forest model also performed well, with AUROC scores of 0.78 for prolonged LOS, 0.87 for CXR prediction, and 0.84 for inpatient admissions. Although the logistic regression model was less accurate overall, it still provided AUROC values of 0.70, 0.79, and 0.74 for the same outcomes, respectively. These findings suggest that ML models offer reliable predictive insights, particularly for frequently ordered investigations like CXR, and hold promise for enhancing clinical workflows.

Key predictors for prolonged ED LOS included terms reflecting the severity of presentation and involvement of emergency services. Notably, the presence of terms such as “SAAS” (South Australian Ambulance Service), “SAPOL” (South Australian Police), and “ITO” (Inpatient Treatment Orders) were strongly associated with extended ED stays.

Our results closely resemble those of Patel et al [1], especially regarding the effective use of traditional and advanced ML techniques as clinical predictors in the ED. Both studies demonstrate the potential benefits of integrating ML into ED workflows. Our research contributes further insights by acknowledging the impact of systemic factors (eg, inpatient bed occupancy on LOS predictions), integration of which could improve the predictive accuracy of ML models and further boost their clinical utility.

Both studies highlight the need for ongoing research into ML application in health care. Future studies should explore the role of systemic factors and real-time data integration to further enhance the clinical utility of ML models in ED settings, ultimately improving patient outcomes and operational efficiency.

## Abbreviations

AUROC: area under the receiver operating characteristic curve
BERT: Bidirectional Encoder Representations from Transformers
CXR: chest x-ray
ED: emergency department
ITO: Inpatient Treatment Orders
LOS: length of stay
ML: machine learning
SAAS: South Australian Ambulance Service
SAPOL: South Australian Police
TF-IDF: term frequency–inverse document frequency
XGBoost: extreme gradient boosting
